# WHO AWaRe classification for antibiotic stewardship: tackling antimicrobial resistance – a descriptive study from an English NHS Foundation Trust prior to and during the COVID-19 pandemic

**DOI:** 10.3389/fmicb.2023.1298858

**Published:** 2023-12-11

**Authors:** Rasha Abdelsalam Elshenawy, Nkiruka Umaru, Zoe Aslanpour

**Affiliations:** Department of Pharmacy, School of Life and Medical Sciences, University of Hertfordshire, Hatfield, United Kingdom

**Keywords:** AWaRe, antibiotic stewardship, COVID-19, NHS, hospitals, antimicrobial resistance

## Abstract

Antimicrobial resistance (AMR) is a silent and rapidly escalating pandemic, presenting a critical challenge to global health security. During the pandemic, this study was undertaken at a NHS Foundation Trust in the United Kingdom to explore antibiotic prescribing trends for respiratory tract infections (RTIs), including pneumonia, and the COVID-19 pandemic across the years 2019 and 2020. This study, guided by the WHO’s AWaRe classification, sought to understand the impact of the pandemic on antibiotic prescribing and antimicrobial stewardship (AMS). The research methodology involved a retrospective review of medical records from adults aged 25 and older admitted with RTIs, including pneumonia, in 2019 and 2020. The application of the AWaRe classification enabled a structured description of antibiotic use. The study evaluated antibiotic use in 640 patients with RTIs. Notably, it observed a slight increase in the use of amoxicillin/clavulanic acid and a substantial rise in azithromycin prescriptions, highlighting shifts in prescribing trends. Despite these changes, some antibiotics displayed steady consumption rates. These findings highlight the importance of understanding antibiotic use patterns during the AMR threat. The increase in the usage of “Watch” category antibiotics during the pandemic emphasises the urgency of robust AMS measures. The research confirms that incorporating the AWaRe classification in prescribing decisions is crucial for patient safety and combating antibiotic misuse. This study provides essential insights into the changing landscape of antibiotic prescribing during a global health crisis, reinforcing the necessity for ongoing AMS vigilance to effectively address AMR challenges.

## Graphical abstract

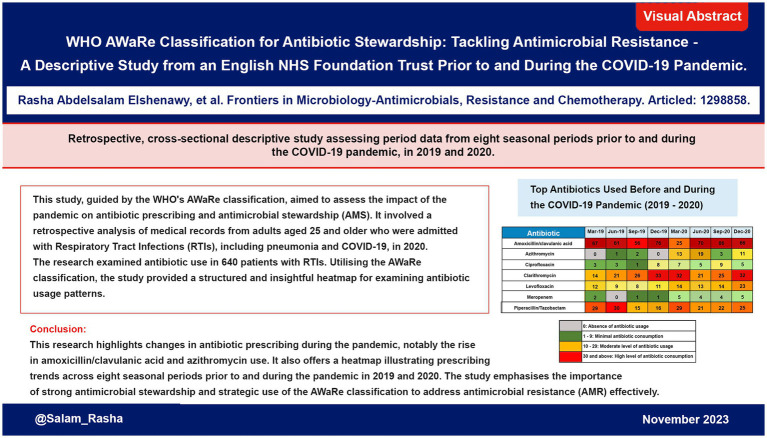

## Introduction

Antimicrobial resistance (AMR) constitutes a silent and rapidly escalating pandemic, presenting a critical challenge to global health security (Salam et al., 2023). The introduction of penicillin in the 1920s was a transformative era in the field of infection management, significantly diminishing mortality rates associated with infections (Elshenawy et al., 2023). Despite these advances, the emergence and escalation of AMR, primarily attributed to the inappropriate prescription of antibiotics, casts a long shadow over these achievements. It was projected that AMR infection rates could escalate to 10 million cases by the year 2050 (O’Neill, 2014). Notably, in 2019, AMR was implicated in the deaths of over 1.2 million individuals globally. In response to this growing crisis, the implementation of antimicrobial stewardship (AMS) has become increasingly imperative (Murray, 2022). AMS advocates for the judicious use of antibiotics, thereby optimising treatment outcomes and minimising the development of resistance (National Institute for Health and Care Excellence, 2015). The World Health Organization (WHO) has actively contributed to this endeavour by developing the AWaRe classification system (www.who.int, 2023). This framework is instrumental in guiding the global implementation of AMS and promoting responsible antibiotic usage, aligning with the strategic objectives outlined in the UK’s Five-Year AMR Strategic Plan (Department of Health and Social Care, 2019). This alignment emphasised an integrated international commitment to addressing and mitigating the challenges posed by AMR (Tejpar et al., 2022).

In the AWaRe tool, antibiotics are divided into three categories: access, watch, and reserve. Each category is based on its respective effect on AMR. The “Access” antibiotics are characterised by their narrow spectrum of activity, typically resulting in fewer side effects, a reduced likelihood of antimicrobial resistance selection, and lower costs. They are strongly recommended for empiric treatment of common infections and should be readily available. Conversely, “Watch” antibiotics carry a higher risk of promoting antimicrobial resistance and are primarily prescribed for patients with more severe conditions, predominantly within hospital settings. Vigilant monitoring of these antibiotics is vital to prevent their overuse. “Reserve” antibiotics, however, are considered the last resort and should be employed only when dealing with severe infections caused by multidrug-resistant pathogens. Their use should be reserved for critical situations. The AWaRe classification underscores the importance of restricting the use of “Watch” and “Reserve” category antibiotics. By 2023, the WHO aims for at least 60% of all antibiotic consumption to come from the Access group (www.who.int, 2023).

The COVID-19 pandemic has profoundly impacted global healthcare systems and various aspects of people’s lives worldwide (World Health Organization, 2021). An inevitable consequence of the pandemic has been the increase in inappropriate antibiotic use, contributing to rising AMR rates (Subramanya et al., 2021). This is despite WHO guidelines advising against antibiotics unless there is strong evidence of a secondary bacterial infection (World Health Organization, 2020). Surprisingly, it was found that 70% of COVID-19 patients were administered antimicrobials (Pérez de la Lastra et al., 2022). Consequently, the inappropriate use of antibiotics during the COVID-19 pandemic may exacerbate the global challenge of AMR (Nandi et al., 2023). This research aimed to examine the use of antibiotics in the initial and subsequent treatment stages of RTIs, including pneumonia, both prior to pandemic (PP) and during the pandemic (DP) at one English National Health Service (NHS) Foundation Trust. In order to provide an in-depth understanding of the impact of the pandemic on antibiotic prescribing, we analysed data from eight seasonal time points in 2019 and 2020.

## Method

### Study design

**Figure 1:**
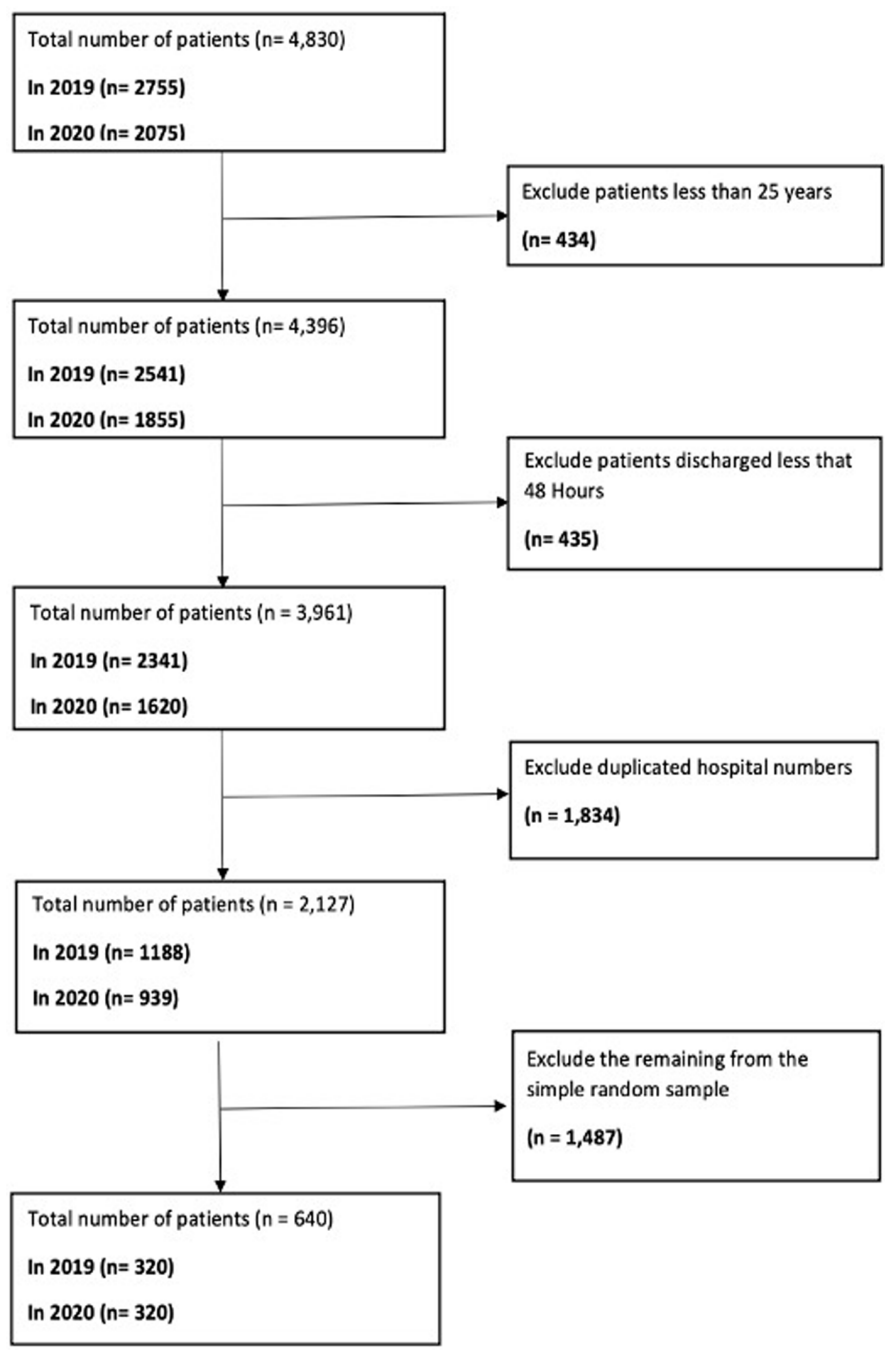
Data filtering algorithm for extracting a representative sample of 640 patient medical records from 2019 and 2020.

A retrospective cross-sectional patient records review study was conducted to investigate AMS and the AWaRe classification of antibiotics in adult patients aged 25 and older. These patients were admitted to an English NHS Foundation Trust in the United Kingdom during 2019 and 2020. The study comprehensively describes antibiotic prescribing patterns utilising a methodological approach based on retrospective cross-sectional analysis. For sampling, the systematic method was employed to consistently select patient medical record data from a larger dataset at the Trust. Initially, data from 4,830 records (2,755 from 2019 and 2,075 from 2020) were extracted. After applying inclusion and exclusion criteria and eliminating duplicate records, the numbers were narrowed down to 1,188 for 2019 and 939 for 2020. Subsequently, a random selection of 80 records for each of the four-time points in 2019, as well as 80 records from 2020, was conducted using Excel’s Random function. This resulted in a total of 640 patient records (as shown in Figure 1). The systematic sampling method ensured equal representation across the patient population and was consistently applied across all eight seasonal time points, spanning from Spring 2019 to Winter 2020. This approach streamlined the sampling process whilst ensuring a comprehensive representation of the patient population.

### Sample size

According to estimations by Public Health England, approximately 20% of antibiotic prescriptions in the UK are potentially inappropriate. For this study, the sample size was determined using Minitab statistical software, taking into account the total population size, a margin of error set at 10%, and a confidence interval of 95%. The data collection involved patient records across eight different seasonal time points spanning 2019 and 2020. The analysis encompassed a total of 640 medical records, divided evenly between 2019 (pre-pandemic period) and 2020 (during the pandemic period), with each year comprising four seasonal time points. To ensure a robust and representative sample, a systematic sampling method was employed to select 80 patients for each of these intervals.

### Study population (inclusion/exclusion criteria)

A stratified sampling strategy was employed to ensure maximum diversity amongst the included medical records. The inclusion criteria comprise the following: (i) adult patients aged 25 years and older; (ii) patients admitted to the Trust; (iii) patients admitted in 2019 and 2020; (iv) patients prescribed antibiotics for RTIs, including pneumonia; and (v) pregnant women and immunocompromised patients. However, patients who spent less than 48–72 h in the Accident and Emergency (A&E) department, patients who were not prescribed antibiotics, and children were excluded from this study.

### Data source

The primary author (RAE) was responsible for gathering data from the patient’s electronic patient records within the Trust, strictly following the inclusion and exclusion criteria established for the study.

### Data collection

Data were collected from the medical records of 640 patients within the Foundation Trust in accordance with the specified inclusion and exclusion guidelines. Data were collected from eight-time points, with four-time points PP: (i) March (Spring 2019); (ii) June (Summer 2019); (iii) September (Autumn 2019); and (iv) December (Winter 2019). Additionally, four-time points occurred DP: (i) March (Spring 2020) – the first wave of COVID-19; (ii) June (Summer 2020) – the first lockdown; (iii) September (Autumn 2020) – the second wave of the pandemic; and (iv) December (Winter 2020) – the vaccination rollout.

### Data extraction

**Figure 2:**
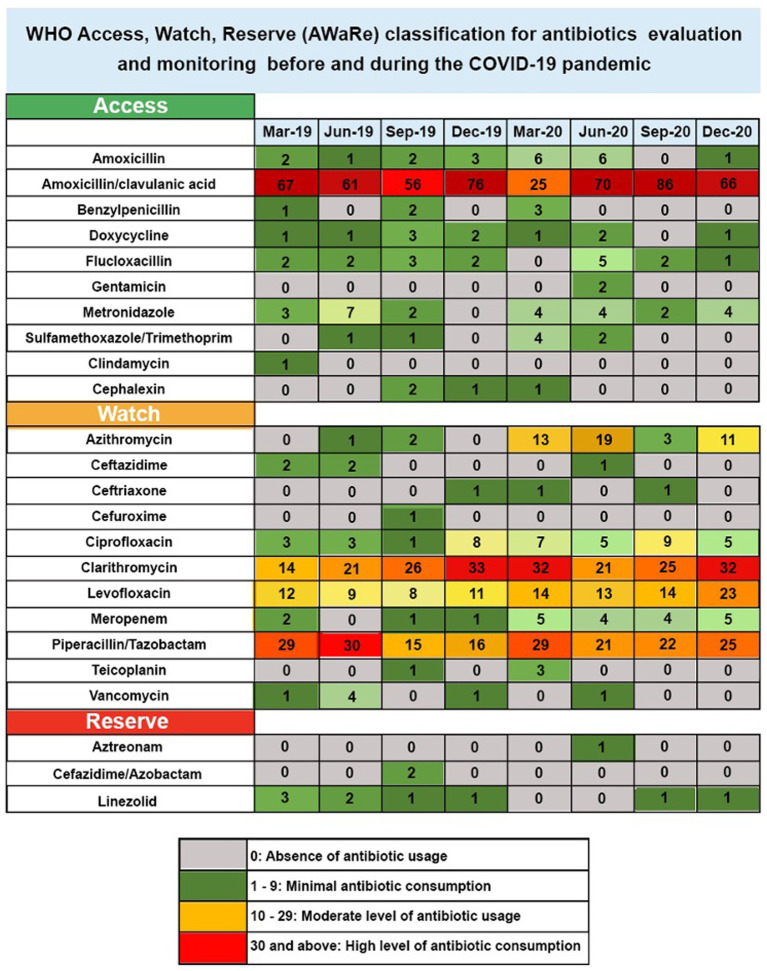
Heatmap for antibiotic use in 2019 and 2020 according to AWaRe criteria.

A data extraction tool was utilised to retrieve essential information from 320 medical records of patients diagnosed with RTIs, such as pneumonia, in the pre-pandemic year of 2019. Additionally, the same tool was used to extract data from another set of 320 records of patients diagnosed with RTIs, including pneumonia and COVID-19, during the pandemic in 2020. The data extraction tool was set up in line with the guidelines of the WHO AWaRe Tool1 (www.who.int, 2023). This study focussed exclusively on antibiotics utilised for treating RTIs. Following the categorisation by the WHO AWaRe Tool, 10 antibiotics were classified under the “Access” group, 11 were identified as “Watch” antibiotics, and 3 were allocated to the “Reserve” category, as shown in Figure 2. The data extraction tool encompassed comprehensive details, including patient demographics, initial diagnosis, and the usage of first- and second-course antibiotics. Adhering to the guidelines of the WHO AWaRe Tool, the primary author dedicated approximately 45 min to each patient’s medical record for the successful extraction of the required data.

### Pilot study

The primary author conducted a pilot study in which data were derived from 10 patient medical records at each time point, accumulating 80 patient medical records in 2019 and 2020. The primary objectives of this pilot study were to provide an initial characterisation of the data and to evaluate the viability of the data extraction instrument in addressing the research queries. Descriptive statistical analysis was employed to interpret this initial data. The findings from this pilot study indicated that the data extraction tool was effective in meeting all the objectives of the study. The data generated and extracted during the pilot phase were not included in the study’s final analysis.

### Validity and reliability of the data extraction tool

The lead author (RAE) developed the data extraction tool using literature sources, the AMS toolkit from Public Health England (PHE), and the World Health Organization’s (WHO) AWaRe Tool. The tool’s components were finalised through collaborative discussions amongst the authors. To validate the tool, RAE and an AMS pharmacist at the research NHS Foundation Trust independently extracted data from 10 medical records at each sample time point. A minimum agreement rate of 80% was set as the standard for tool validation. Additionally, to assess the tool’s reliability, RAE and the AMS pharmacist separately conducted data extractions from the same set of samples. The inter-rater reliability was measured by the percentage of agreement in their independently extracted data, with any variances resolved through joint discussions.

### Patient characteristics

Data were extracted from patient medical records for those admitted in 2019 and 2020, specifically focussing on patients diagnosed primarily with RTIs, including pneumonia, and COVID-19 positive cases, as COVID-19 has the potential to cause secondary bacterial infections that necessitate antibiotic treatment. For patients admitted in 2020, during the pandemic, cases of COVID-19 were also included in the data extraction. The selection of primary RTI diagnoses was guided by the pertinent categories in the International Classification of Diseases, 10th Revision (ICD-10). The extracted data focussed on antibiotics given at admission, empirical antibiotic treatments, and antibiotics prescribed within 48–72 h or 5–7 days following admission.

### Patient and public involvement

The study protocol was submitted to the Citizens Senate, an organization focussed on patient care with a considerable representation of elderly individuals. They provided useful suggestions and comments.

### Registration

This study has been officially registered with the ISRCTN registry. The ISRCTN registry is a primary registry acknowledged by the WHO and the International Committee of Medical Journal Editors (ICMJE), accepting all clinical research studies (www.isrctn.com, 2022). Moreover, it was registered in Octopus, the global primary research record (Octopus, 2019).

### Data analysis

This study identified the initial antibiotic prescribed for each patient according to the main diagnosis of RTIs, PP, and DP. Additionally, AMS was evaluated using the AWaRe classification, descriptive statistics, and data analysis software, Excel 2019 for Windows (www.microsoft.com, 2019).

## Results

### WHO AWaRe tool: antibiotic usage in RTIs

This research examined the antibiotics prescribed for RTIs in 640 patients admitted between 2019 and 2020. In the Figure 1 heatmap, each row represents a different antibiotic, whilst each column corresponds to a specific seasonal month from 2019 to 2020. The colour intensity of each cell in the heatmap represents the frequency of prescriptions for each antibiotic used in treating RTIs, including pneumonia and COVID-19-positive cases, across the 640 patients admitted during those years. This visualization is particularly informative given that COVID-19 can lead to secondary bacterial infections requiring antibiotic intervention. Darker colours indicate higher prescription rates, providing a visual representation of prescribing trends over time. The heatmap uses a colour-coded system to reflect the levels of antibiotic consumption over several months, from March 2019 to December 2020. Antibiotics are categorised into three groups based on the World Health Organization’s Access, Watch, and Reserve (AWaRe) classification, which is designed to promote the proper use of antibiotics to combat resistance. In this heatmap figure, antibiotic consumption was categorised into four levels based on values, with the highest value being 86 and the lowest value being 0. These categories were as follows: 0 represented the absence of antibiotic usage, 1–9 represented minimal antibiotic consumption, 10–29 represented a moderate level of antibiotic usage, and 30 and above represented the highest level of antibiotic consumption (Figure 2). The categorisation of data in Figure 2 is derived from a literature review and the clinical relevance of antibiotic prescribing trends.

The “Access” category includes essential antibiotics that should be widely available. In this category, amoxicillin/clavulanic acid showed a substantial increase, starting at 67 in March 2019 and peaking at 86 in September 2020, indicating high usage. Flucloxacillin also demonstrated an increase from 2 in March 2019 to 5 in June 2020, suggesting moderate use. The “Watch” group comprises antibiotics that have the higher potential for resistance and should be used more cautiously. In this study, azithromycin usage escalated from zero in March 2019 to 19 in June 2020, showing a high level of use. Clarithromycin started at 21 in June 2019 and increased to 32 by December 2020. Ciprofloxacin and levofloxacin also saw increases in their consumption levels over the study period. Piperacillin/tazobactam maintained a consistently high consumption level of 29 in March 2019 and March 2020. Meropenem showed a modest increase from 2 in March 2019 to 5 in December 2020. In the “Reserve” category, which includes antibiotics that should be reserved for treating infections caused by multidrug-resistant organisms, linezolid maintained a high consumption level of 3 in March 2019 without any substantial increase through 2020. Aztreonam and ceftazidime/avibactam show minimal increases in usage.

Notably, there was an increase in the total usage of antibiotics in the “Access” category, reaching 305 in 2019 and slightly decreasing to 298 in 2020. In contrast, the “Reserve” category saw a reduction in use, declining from 9 to 3. Meanwhile, the “Watch” category experienced a significant increase in 2020, with usage escalating to 386, up from 259 in the previous year.

### The top seven prescribed antibiotics before and during the COVID-19 pandemic

**Figure 3:**
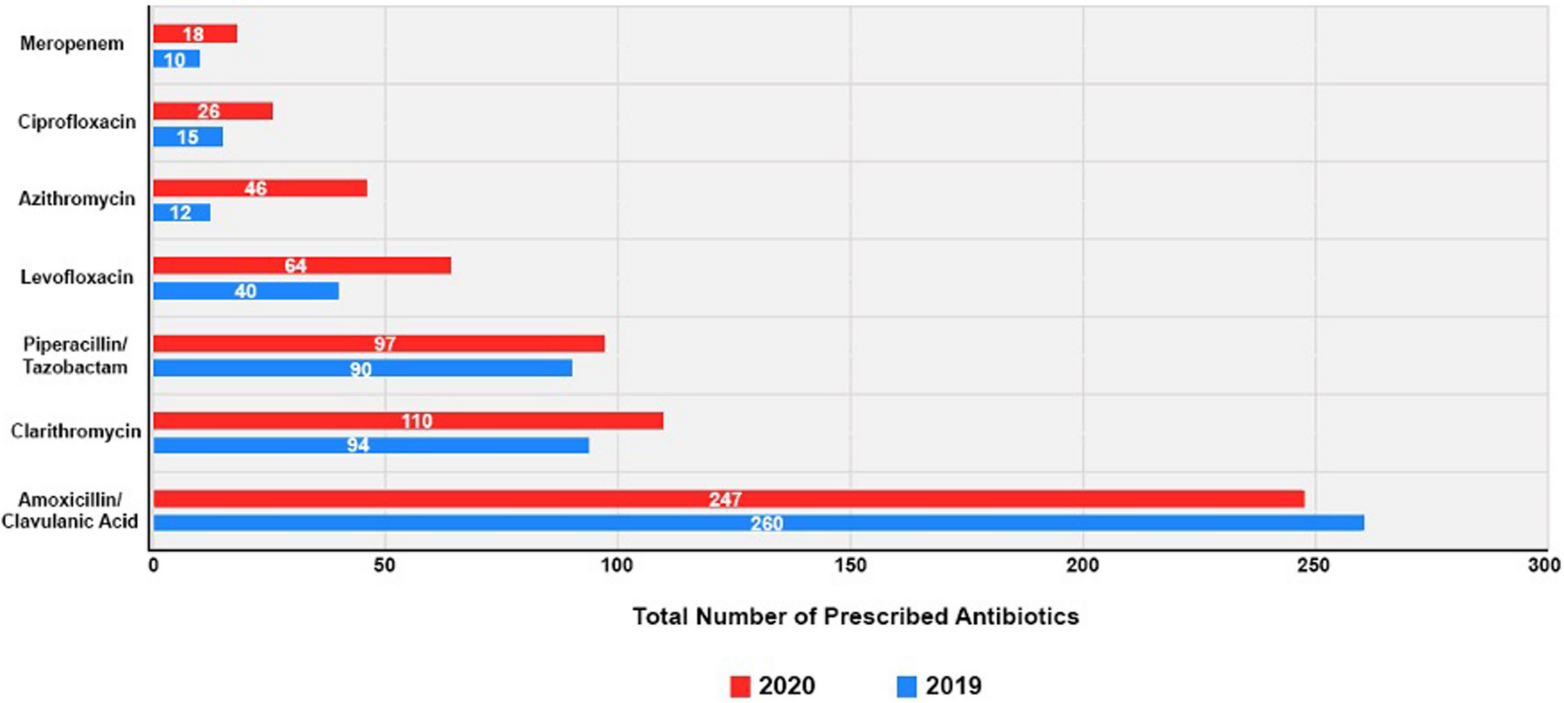
Seven commonly prescribed antibiotics prior to and during the COVID-19 pandemic.

Figure 3 shows the use of the seven most commonly prescribed antibiotics in both PP and DP, further detailed in Supplement 1. In 2019, amoxicillin/clavulanic acid was the most frequently prescribed antibiotic, accounting for 247 instances. This trend persisted in 2020 with 260 instances, maintaining its top position. In 2020, compared to 2019, there was an increase in prescriptions for most of the other antibiotics. For instance, clarithromycin saw an increase from 94 prescriptions in 2019 to 100 in 2020. Piperacillin/tazobactam also witnessed a slight increase, from 90 instances in 2019 to 97 in 2020. Additionally, 2020 showed increased prescriptions of levofloxacin, azithromycin, and ciprofloxacin compared to 2019. Levofloxacin prescriptions increased from 40 in 2019 to 64 in 2020. Azithromycin had a surge, increasing from 12 in 2019 to 46 in 2020. Ciprofloxacin also displayed a increasing trend, going from 15 in 2019 to 26 in 2020, whilst meropenem’s usage modestly increased in 2020, from 10 to 18 instances.

## Discussion

This study examined the prescribing patterns of antibiotics at an English NHS Foundation Trust, employing the AWaRe classification system for antibiotics used in treating RTIs, including pneumonia, and taking into account cases positive for COVID-19 in the year 2020. This AWaRe classification serves as an effective means for tracking antibiotic usage, establishing goals, and observing the impact of stewardship initiatives aimed at enhancing antibiotic utilisation and combating antimicrobial resistance. The WHO’s 13th General Programme of Work for the years 2019–2023 sets a target for countries to achieve at least 60% of their total antibiotic consumption from antibiotics categorised in the access group (www.who.int, 2021). COVID-19 highlighted the effectiveness of antimicrobial stewardship (AMS) in combating AMR by guiding strategic choices during the pandemic and encouraging judicious antibiotic use. Findings from this study showed that amoxicillin/clavulanic acid, classified in the “Access group,” emerged as the most frequently prescribed antibiotic (Elshenawy et al., 2023).

This study observed a significant increase in the use of azithromycin, an antibiotic categorised in the “Watch” group. This categorisation indicates a need for more cautious use due to a higher potential for resistance development. This upward trend in azithromycin usage aligns with findings from a variety of international research efforts. In India, studies by Mugada et al. (2021) and Sharma et al. (2021) reported similar patterns, reflecting a broader shift in antibiotic prescription practices. In Malaysia, Mohamad et al. (2022) also observed an increase. In Zambia, findings by Mudenda et al. (2022, 2023) further corroborate these findings, suggesting both a regional and a global increase in reliance on azithromycin. Additionally, research conducted in several Eastern Mediterranean countries, including studies by Kalungia et al. (2022) and Jirjees et al. (2022), and in other regions such as Ghana, as noted by Amponsah et al. (2022), has also observed this trend, highlighting a consistent global shift towards increased usage of this particular antibiotic. These studies collectively emphasise the growing preference for azithromycin across diverse geographical and clinical settings (Mugada et al., 2021; Sharma et al., 2021; Amponsah et al., 2022; Jirjees et al., 2022; Kalungia et al., 2022; Mohamad et al., 2022; Mudenda et al., 2022; Mudenda et al., 2023).

Possible reasons for this increase could be the rise in respiratory tract infections, including pneumonia, suspected to be COVID-19 during the pandemic, as well as the early inclusion of antibiotics, such as azithromycin, in COVID-19 treatment protocols (Mugada et al., 2021). A study conducted in Zambia in 2022 analysed antibiotic prescriptions (443 instances) and found that ceftriaxone and metronidazole were the most commonly prescribed antibiotics. A total of 42.1% of antibiotics in the “Watch” category exceeded recommended limits, emphasising the urgent need for improved antimicrobial stewardship and adherence to guidelines (Mudenda et al., 2023). An increasing trend in the use of antibiotics categorised in the “Watch group” has been observed in this study. This trend may stem from concerns regarding the effectiveness of “Access group” antibiotics, their availability, and patient demands. It highlights the necessity for enhanced availability of “Access group” antibiotics and a reduction in the utilisation of “Watch group” antibiotics, which are more prone to resistance.

In the “Watch” category, antibiotic use significantly increased during the COVID-19 pandemic in 2020, totalling 386 compared to 259 in the pre-pandemic year of 2019. This trend illuminates evolving prescribing practices and highlights the necessity of enhanced antibiotic stewardship. The study also revealed that antibiotics in the “Watch” group were the most used, accounting for 45.8% of the total, with the “Access” group following at 42.7% and the “Reserve” group comprising 12.5% of the usage. In Ghana, a study conducted in 2022 revealed that there was a notable inclination towards prescribing antibiotics from the “Access” group. This trend reflects specific regional prescribing patterns and underscores the dominant role of “Access” group antibiotics in these countries’ healthcare practices. In India, antibiotic use was compared over 2 years using the AWaRe index tool. The study, retrospective in nature, analysed data from January 2017 to December 2018. Results showed a shift in antibiotic consumption: in 2017, 53.31% of antibiotics used were from the “Access” category, 40.09% from “Watch”, and 3.40% from “Reserve”. In 2018, these figures changed to 41.21, 46.94, and 8.15%, respectively, indicating a 17% increase in “Watch” and 140% in “Reserve” category usage, suggesting evolving resistance (Sharma et al., 2021). The study revealed that in 2020, during the COVID-19 pandemic, there was a substantial increase in the usage of levofloxacin, azithromycin, and ciprofloxacin compared to their consumption levels in 2019, highlighting a shift in antibiotic prescribing patterns during the health crisis.

Analysing the impact of the COVID-19 pandemic on antibiotic prescribing patterns within the UK NHS Trust during 2019 and 2020 presents a multifaceted challenge. The study indicates an increased use of specific antibiotics such as levofloxacin, azithromycin, and ciprofloxacin in 2020, which might be linked to the treatment approaches and uncertainties prevalent in the early stages of the pandemic. Additionally, the pandemic led to notable shifts in healthcare practices, potentially influencing these prescribing behaviours. However, the study’s retrospective and cross-sectional nature may not fully capture all the confounding factors, such as the severity of infections or varied patient health conditions. The data, whilst indicative, might not comprehensively represent all cases or the complete spectrum of clinical decision-making, making it difficult to precisely quantify the direct impact of the pandemic on antibiotic prescribing patterns. This study is descriptive, providing a visual tool to observe antibiotic prescriptions in 2019 and 2020, as well as to visualise how the COVID-19 pandemic impacted antibiotic prescribing. It also identifies areas requiring AMS implementation. Additionally, identify the areas required for AMS implementation. Although estimating patient days was vital, it presented challenges in this study, necessitating further research on this aspect.

### Limitations

The study has several limitations. It excludes children under 12 years old and faces challenges in accurately calculating patient days, potentially affecting the evaluation of antibiotic use compared to patient volume. The study’s focus on a limited number of prescriptions may not encompass the entire range of prescribing behaviours. As a retrospective and cross-sectional analysis, it has limitations in considering the varied health statuses and treatment reactions of patients and may not effectively measure compliance with clinical prescribing guidelines. These factors indicate that the study might not provide a comprehensive assessment of the impact of antibiotic prescribing on patient outcomes.

## Conclusion

This study provides valuable insights into the dynamics of antibiotic prescribing within a UK NHS Foundation Trust during the COVID-19 era. Analysing data from 640 patients, it reveals shifts in antibiotic use for respiratory infections, including pneumonia and COVID-19, across four seasonal phases. Key findings reveal a progressive increase in the use of amoxicillin/clavulanic acid in the Access category from March 2019 to September 2020. In the Watch category, there was a notable increase in the consumption of antibiotics such as azithromycin, clarithromycin, ciprofloxacin, and levofloxacin within the same timeframe. Piperacillin/tazobactam usage remained consistently high, whilst meropenem saw a slight uptick. This pivotal study not only traces the evolution of prescribing practices during the pandemic but also highlights the critical need for vigilant antimicrobial stewardship to combat resistance and safeguard patient health.

## Data availability statement

The datasets presented in this article are not readily available because this data is restricted and confidential with the institution policy. Requests to access the datasets should be directed to r.a.elshenawy@herts.ac.uk.

## Ethics statement

The studies involving humans were approved by Ethical approval for this study was granted by the Health Research Authority (HRA), with the Research Ethics Committee (REC) assigning reference number 22/EM/0161. In compliance with this approval, the study protocol underwent review and received approval from the University of Hertfordshire (UH) ethics committee under the reference LMS/PGR/NHS/02975. The authors have no conflicts of interest to disclose. The studies were conducted in accordance with the local legislation and institutional requirements. Written informed consent for participation was not required from the participants or the participants’ legal guardians/next of kin in accordance with the national legislation and institutional requirements.

## Author contributions

RE: Formal analysis, Investigation, Methodology, Validation, Visualization, Writing – original draft. NU: Supervision, Visualization, Writing – review & editing. ZA: Supervision, Visualization, Writing – review & editing.
