# Supplementary figures and images for: WHO AWaRe classification for antibiotic stewardship: tackling antimicrobial resistance – a descriptive study from an English NHS Foundation Trust prior to and during the COVID-19 pandemic
Source: Front Microbiol. 2023 Dec 11;14:1298858. doi: 10.3389/fmicb.2023.1298858 (PMC10749484; doi:10.3389/fmicb.2023.1298858)

**Supplement 1.** Top Antibiotics Utilized Before and During the COVID-19 Pandemic in 2019 and 2020


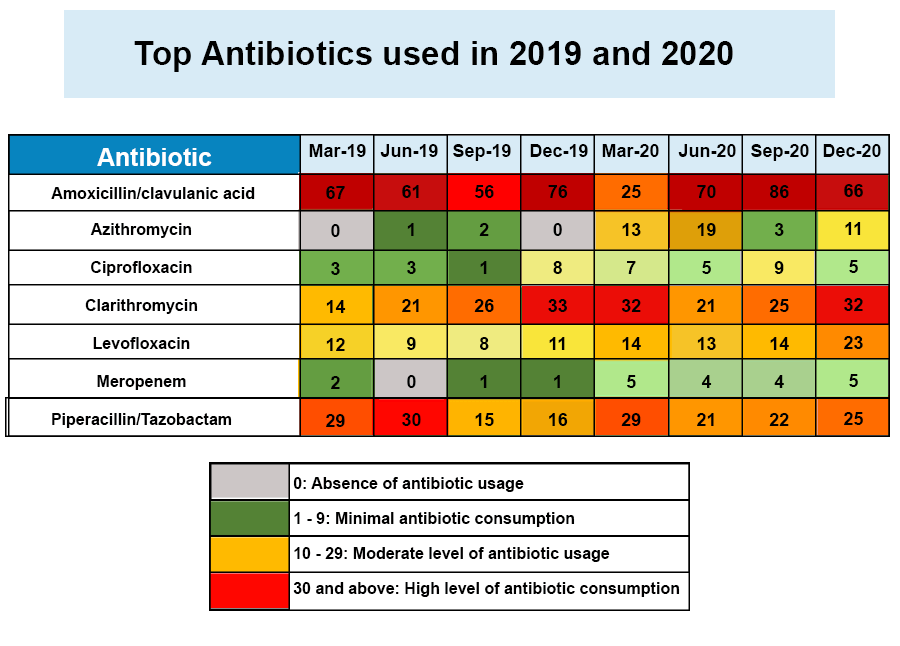

Supplement: Supplementary file 1 [file Table_1.DOCX]
